# Flat Photonic Crystal Fiber Plasmonic Sensor for Simultaneous Measurement of Temperature and Refractive Index with High Sensitivity

**DOI:** 10.3390/s22239028

**Published:** 2022-11-22

**Authors:** Wei An, Chao Li, Dong Wang, Wenya Chen, Shijing Guo, Song Gao, Chunwei Zhang

**Affiliations:** 1 School of Information Science and Engineering, University of Jinan, Jinan 250022, China; 2 Shandong Provincial Key Laboratory of Network-Based Intelligent Computing, Jinan 250022, China

**Keywords:** photonic crystal fiber, surface plasmon resonance, refractive index sensor, temperature sensor, dual-parameter measurement

## Abstract

A compact temperature-refractive index (RI) flat photonic crystal fiber (PCF) sensor based on surface plasmon resonance (SPR) is presented in this paper. Sensing of temperature and RI takes place in the *x*- and *y*- polarization, respectively, to avoid the sensing crossover, eliminating the need for matrix calculation. Simultaneous detection of dual parameters can be implemented by monitoring the loss spectrum of core modes in two polarizations. Compared with the reported multi-function sensors, the designed PCF sensor provides higher sensitivities for both RI and temperature detection. A maximum wavelength sensitivity of −5 nm/°C is achieved in the temperature range of −30–40 °C. An excellent optimal wavelength sensitivity of 17,000 nm/RIU is accomplished in the RI range of 1.32–1.41. The best amplitude sensitivity of RI is up to 354.39 RIU^−1^. The resolution of RI and temperature sensing is 5.88 × 10^−6^ RIU and 0.02 °C, respectively. The highest value of the figure of merit (FOM) is 216.74 RIU^−1^. In addition, the flat polishing area of the gold layer reduces the manufacturing difficulty. The proposed sensor has the characteristics of high sensitivity, simple structure, good fabrication repeatability, and flexible operation. It has potential in medical diagnosis, chemical inspection, and many other fields.

## 1. Introduction

Optical fiber sensing technology has great potential for development in bio-chemical detection, environmental monitoring, medical diagnosis, and many more fields [1,2,3,4,5,6] due to its small size, low cost, anti-electromagnetic interference, and structural stability. Photonic Crystal Fiber (PCF) is composed of core and cladding with periodically arranged air holes that control the propagation of light. The flexible geometry arrangement of the PCF enables it to provide desirable characteristics [7]. In addition, the air holes can be selectively filled with materials such as thermo-sensitive liquid and analyte to realize various parameter sensing. Compared with traditional optical fibers, PCF sensors combined with surface plasmon resonance (SPR) have the advantages of real-time detection, flexible design, and high sensitivity. With the development of SPR-PCF sensors, the detection of a single parameter, such as refractive index (RI), temperature, etc., has gradually grown into maturity. A D-shaped PCF-SPR sensor proposed by Meng X et al. [8] shows a double confinement loss (CL) peak and an unchangeable spectrum trough that indicates the extreme stability of the sensor. Siddik A B et al. [9] reported a dual-core PCF temperature sensor. Under the temperature range of 0–60 °C, the sensor shows a flat sensitivity of 2.25 nm/°C.

However, if the detection of multiple parameters is desired, operators need to cascade several single-parameter sensing devices. The complex setup greatly increases the difficulty of operation as well as the manufacturing cost. Therefore, multi-parameter sensors were developed. Many studies have reported possible structures for simultaneous measurement of two or more parameters such as RI, temperature, magnetic field, seawater salinity, strain, voltage, etc. By filling the PCF structure with materials that are sensitive to different parameters in different positions, the simultaneous sensing of multiple parameters can be realized in multiple channels. The D-shaped PCF proposed by Zhang Y et al. [10] realizes simultaneous sensing of the RI and seawater temperature. Calculating the wavelength sensitivity of the RI and temperature, the maximum values of 1228 nm/RIU and −1.06 nm/°C were obtained, respectively. In 2018, Yong et al. [11] proposed a C-type grapefruit micro-structured fiber for the measurement of seawater temperature and salinity, with a maximum response sensitivity of 1.402 nm/‰ and −7.609 nm/°C for salinity and temperature, respectively. The detection of multiple parameters often has the problem of cross-sensitivity, which increases the difficulty of separating multiple signals. The proposed structure above uses the transfer matrix to express metrics such as sensitivity, resolution, etc., of each parameter, which makes the processing of parameters complicated. The D-shaped RI and temperature sensor proposed by Chen A et al. [12] separates the detection of the two parameters in the *x*- and *y*-polarizations, respectively, and avoids the crossover of signals. However, the obtained maximum sensitivity is only 3940 nm/RIU and 1.075 nm/°C, respectively. The simple D-shaped coreless fiber sensor based on SPR proposed by Li B et al. [13] can measure the liquid RI and ambient temperature in real-time. The optimal sensitivity reaches 12,530 nm/RIU and −3.465 nm/°C, respectively. The above configuration of structures is comparatively concise. Nevertheless, the acquired sensitivity is unideal. In conclusion, improving the sensing performance of the structure design is required.

In this paper, we propose and illustrate a compact RI-temperature PCF plasmonic sensor. The RI detection channel is designed as a flat surface and coated with a gold layer. The temperature detection channel is coated with a gold layer and filled with toluene to improve the sensitivity of temperature detection. Separated sensing in *x*- and *y*-polarization of dual-parameter averts sensitivity crossover. Simultaneous detection is implemented simply by monitoring the polarized guiding light direction. The optimized structure accomplishes the maximum wavelength sensitivity of −5 nm/°C in the temperature range of −30–40 °C. The resonance wavelength of temperature sensing shows great linearity. An excellent optimal wavelength sensitivity of 17,000 nm/RIU is achieved in the RI range of 1.32–1.41. The proposed sensor shows an ideal detecting resolution of 0.02 °C and 5.88 × 10^−6^ RIU for temperature and RI, respectively. The best amplitude sensitivity can be up to 354.39 RIU^−1^. The highest value of the figure of merit (FOM) is 216.74 RIU^−1^. The fabrication tolerance and manufacturing process of the sensor are also investigated in the paper. Owing to its high sensitivity, flexible operation, and superior performance, the sensor exhibits great potential in environmental monitoring, chemical inspection, and many other fields.

## 2. Theory and Model Design

**Figure 1:**
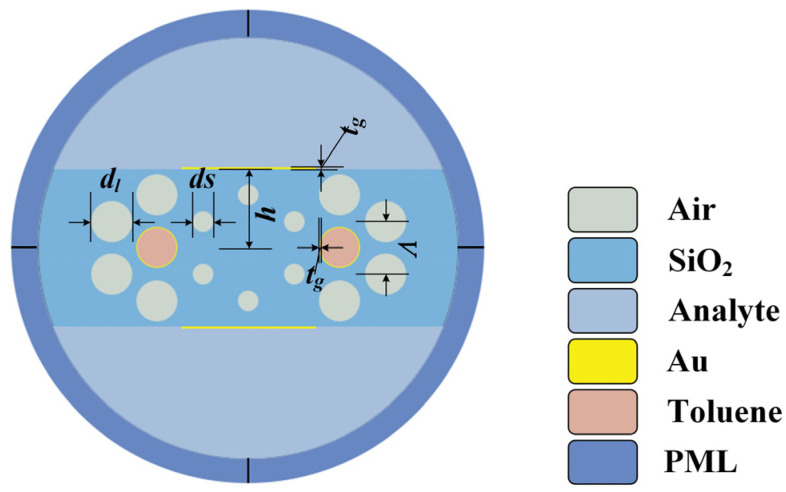
Schematic diagram of the proposed flat PCF-SPR sensor.

A cross-section view of the proposed PCF sensor is shown in Figure 1. The air holes of PCF are composed of a three-layer hexagonal arrangement, and the interval of adjacent air holes is mentioned as pitch, which is denoted by *Λ*. The air hole in the core area is omitted. Therefore, the light wave propagates in the fiber core according to the principle of total internal reflection (TIR) theory. The diameter *d_s_* of the innermost air holes is small, while the diameter *d_l_* of the air holes in the outer two layers is relatively large. Ten air holes in the upper and lower parts, respectively, are omitted for the deposition of the metal film and analyte filling. The gold layer is deposited at a distance *h* from the fiber core to implement the detection of RI. Analyte with variable RI is filled in the upper and lower D-shaped regions in the fiber structure. Analyte liquid is in direct contact with the gold film, achieving the sensitive detection of RI. The thickness of the gold layer is marked as *t_g_*. The flat polishing of gold film greatly reduces the difficulty of manufacturing. In addition, the two air holes that are close to the fiber core in the second layer are deposited with a gold film; the thickness is also *t_g_*. The temperature-sensitive liquid toluene is filled in the two air holes to accomplish the real-time sensitive detection of the ambient temperature. This structural design enables the sensing of the analyte RI and the ambient temperature to be performed in *y*-polarization and *x*-polarization, respectively, avoiding the crossover of the dual parameter detection. The optimized structural parameters are *d_s_* = 0.9 μm, *d_l_* = 1.6 μm, *Λ* = 2 μm, *h* = 3.1 μm, and *t_g_* = 50 nm. We analyze the sensing characteristics of the sensor by monitoring incident light wavelength, the step of which is set as 5 nm. Theoretical simulation of the proposed structure is implemented by the finite element method (FEM). In order to make the unwanted modes leak from the fiber cladding and further diminish the leakage loss, a 1-μm-thick Perfect Matching Layer (PML) is used outside the PCF as an absorbing boundary condition. In addition, the scattering boundary condition is configured in order to better replicate the external environment. Free triangular mesh is used to divide the proposed sensor structure. The model is divided into 27,878 mesh elements in total, with 202,427 degrees of freedom.

**Figure 2:**
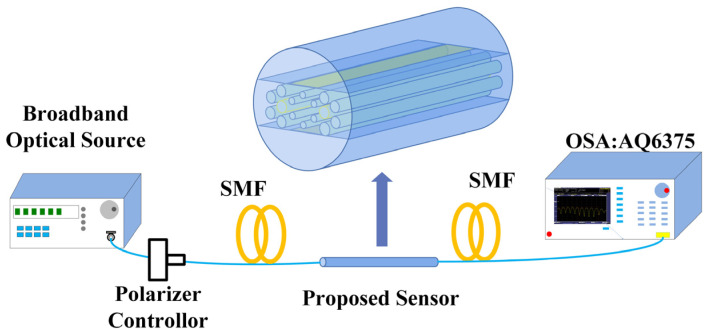
Schematic setup of the flat PCF plasmonic sensor.

Figure 2 shows the experimental setup of the microsensor. A broadband light source (BBS) emits a light signal in the wavelength range of 500–1500 nm. The output signal passes through the polarizer controller. A polarized light signal is then transmitted to the designed sensor through a single-mode fiber (SMF). Part of the light energy is lost in the interaction with the analyte. The splicing method can be employed to couple the proposed PCF with SMF. Filling and removal of the analyte can be accomplished using a microinjecting pump. The remaining optical signal is transmitted into another SMF, which is coupled to the optical spectrum analyzer (OSA). The spectrum is recorded and presented by the OSA.

The material RI of the sensor component varies with different temperatures, causing a removal in the phase matching conditions. The resonance wavelength changes between the RI of the core-guided mode and the SPP mode. This variation is reflected in the absorption spectrum. By observing the loss spectrum, sensitive temperature and RI sensing are implemented. The RI of the PCF background material fused silica can be derived from Sellmeier’s Equation [14],

(1) $$n\left(\lambda \right)=\sqrt{A+\frac{B}{(1-C{/\lambda }^{2})}+\frac{D}{(1-E{/\lambda }^{2})}}$$

where λ is the wavelength of the current operating light in a vacuum environment. *A* = 1.31552 + 0.690754 × 10^−5^ *T*, *B* = 0.788404 + 0.235835 × 10^−4^ *T*, *C* = 0.0110199 + 0.584758 × 10^−6^ *T*, *D* = 0.91316 + 0.548368 × 10^−6^, and *E* = 100.

The free electron gas model of the Drude model is used to describe the movement law of electrons inside of the metal, and the complex dielectric constant of gold is computed as [15]:

(2) $$\varepsilon \left(\omega \right)=\varepsilon_{1}+i\varepsilon_{2}=\varepsilon_{\infty }-\frac{\omega_{p}^{2}}{\omega \left(\omega +i\omega_{c}\right)}$$

where $\varepsilon_{\infty }=9.75$ is the dielectric constant of Au at infinite frequency, $\omega_{p}=1.36\times 10^{16}$ is the plasma frequency of gold in units of rad/s, and $\omega_{c}=1.45\times 10^{14}$ is the collision frequency at $T_{0}=298.15\;K$ [16]. The optical properties of metal are determined by plasma frequency [17] and collision frequency [18,19].

Considering the effect of temperature change on the thickness of the metal layer, Equation (3) is introduced [20]:

(3) $$t_{g}=t_{g0}\left[1+\gamma^{\prime }(T-T_{0})\right]$$

where *t_g*0*_* is the thickness of the gold film at room temperature $T_{0}=298.15K$. *γ*′ is the corrected thermal expansion coefficient and is derived from:

(4) $$\gamma^{\prime }=\gamma \frac{1+\mu }{1-\mu }$$

where the linear expansion coefficient *γ* = 1.42 × 10^−5^ K^−1^, *μ* = 0.42 is the Poisson’s number of the film material. *γ* is replaced by *γ*′, for the expansion of the gold layer is only in the normal direction.

Liquid toluene is chosen as the temperature detection material for its large thermo-optical coefficient and is selectively filled into the air holes in the second layer that are close to the fiber core. RI of the liquid toluene $n_{Toluene}$ can be expressed as [21]:

(5) $$n_{Toluene}=n_{Toluene}\left|{}_{T_{0}=20{}^{\circ}C}\right.+\alpha_{Toluene}\times (T-T_{0})$$

where the thermal-optical coefficient of the RI for toluene $\alpha_{Toluene}=-5.273\times 10^{-4}$ [22]. The RI of toluene at *T*_0_ = 20 °C can be written as [23]:

(6) $$n_{Toluene}=1.474775+6990.31/\lambda^{2}+2.1776\times 10^{8}{/\lambda }^{4}$$

The CL of the core mode is determined to analyze the sensing performance, which strongly relates to the imaginary part of RI. CL can be calculated as follows [24]:

(7) αloss=8.686×2πλImneff×104dB/cm

where Imneff refers to the imaginary part of mode RI. By analyzing the dependence of RI and mode CL, the transmission mechanism of the designed PCF-SPR sensor can be revealed.

## 3. Results and Discussion

**Figure 3:**
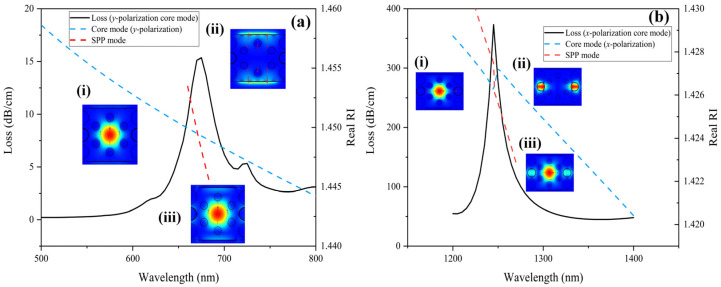
Dispersion relationship of (**a**) *y*- and (**b**) *x*-polarization core modes with SPP modes. The inset (i) (ii) (iii) is the electric field distribution and direction of core modes, SPP modes, and SPR, respectively.

Figure 3a,b represent the dispersion relationship of core modes in *y*- and *x*-polarization with SPP modes. The solid black line is the CL of the mode propagating in the core, the blue dotted line in Figure 3a,b is the real part of the core mode RI in *y*- and *x*-polarization, respectively, and the red dotted line is the real part of the SPP mode RI. It can be seen from Figure 3 that when the RI of the core mode and the SPP mode is equal, the SPR is excited, and a peak appears in the CL spectrum. Inset (i) is the electric field distribution and direction when the core mode is well confined in the core and the CL is relatively low. Inset (ii) is the electric field distribution and direction of the SPP mode in the two detecting channels. As shown in inset (iii), when SPR occurs, the energy of the core mode is transferred to the SPP mode at the resonance wavelength. When the analyte RI or the ambient temperature changes, the position of the resonance wavelength shifts accordingly. Sensitive detecting of the dual parameter can be achieved by observing the change in resonance wavelength. As is illustrated in Figure 3, under RI = 1.36, *T* = 20 °C, in *y*-polarization and *x*-polarization, the core mode and the SPP mode excite SPR at the wavelength of 675 nm and 1245 nm, respectively.

### 3.1. RI Sensing

**Figure 4:**
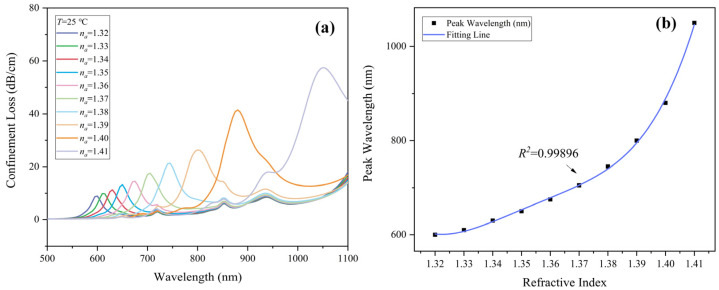
(**a**) CL spectrum of *y*-polarization core mode with various *n_a_* from 1.32–1.41 at *T* = 25 °C. (**b**) Resonance wavelength and its polynomial fitting line with various *n_a_* from 1.32–1.41 at *T* = 25 °C.

The peaks of the CL in *y*-polarization are used to probe the RI change of the analyte. By observing the position of the CL spectrum peak, the analyte RI can be determined accordingly. In Figure 4a, as the analyte RI increases, the position of the CL attenuation peak shifts to a larger wavelength, indicating a red-shift. In order to evaluate the sensitivity of the reported sensor, the wavelength interrogation method is introduced. Wavelength sensitivity is expressed as [25]:

(8) $$S_{\lambda }\left(na,\lambda \right)=\frac{\Delta \lambda_{\mathrm{peak}}}{\Delta n_{a}}\left(\mathrm{nm}/\mathrm{RIU}\right)$$

where $\Delta \lambda_{\mathrm{peak}}$ refers to the shift of peak wavelength under adjacent analyte RIs. The variant of analyte RI is denoted by $\Delta n_{a}$. At *T* = 25 °C, the sensor achieved an optimal wavelength sensitivity of 17,000 nm/RIU when the analyte RI varies from 1.40 to 1.41, which is superior to the prior results among sensors in this category. Furthermore, when the analyte RI changes from 1.32 to 1.40 in steps of 0.01, SPR occurred at 600, 610, 630, 650, 675, 705, 745, 800, and 880 nm, respectively, corresponding to sensitivities of 1000, 2000, 2000, 2500, 3000, 4000, 5500, and 8000 nm/RIU. As shown in Figure 4b, taking the phase matching (resonance) points of core mode and SPP mode, polynomial fitting gives the extremely high fitting coefficient *R*^2^ of 0.99896.

Resolution describes the minimum indices change that a sensor can resolve. The resolution of RI is defined as [26]:

(9) $$R_{\mathrm{RI}}\left(n_{a},\lambda \right)=\Delta n_{a}\times \Delta \lambda_{\min}/\Delta \lambda_{\mathrm{peak}}\left(\mathrm{RIU}\right)$$

where $\Delta n_{a}$ is the change in the adjacent analyte RI, $\Delta \lambda_{\min}$ represents the smallest spectrum resolution, which is usually set as 0.1 nm, $\Delta \lambda_{\mathrm{peak}}$ refers to the shift wavelength of the resonance peak. According to Equation (9), the resolution is 5.88 × 10^−6^ RIU when analyte RI alters from 1.40 to 1.41.

**Figure 5:**
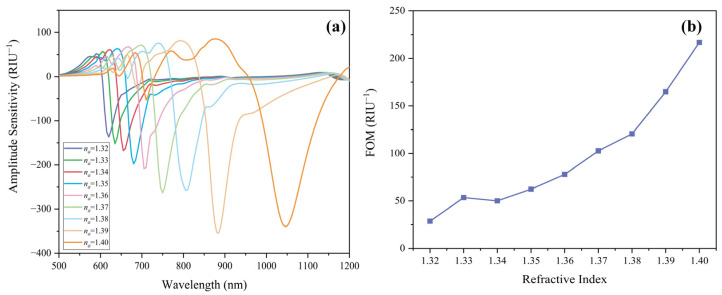
(**a**) Amplitude sensitivity and (**b**) figure of merit with analyte RI changing from 1.32 to 1.41.

Similarly, the amplitude sensitivities of the flat PCF sensor for *n_a_* varies from 1.32 to 1.40 are calculated, and the results are plotted and depicted in Figure 5a. Amplitude sensitivity can be obtained by [27]:

(10) $$S_{A}\left({\mathrm{RIU}}^{-1}\right)=-\frac{1}{\alpha \left(\lambda ,n_{a}\right)}\frac{\partial \alpha \left(\lambda ,n_{a}\right)}{\partial n_{a}}$$

where $\alpha \left(\lambda ,n_{a}\right)$ indicates the mode CL, $\partial \alpha \left(\lambda ,n_{a}\right)$ refers to the difference of CL between the adjacent analyte RI. Amplitude sensitivity reaches a maximum value of 354.39 RIU^−1^ at 885 nm when RI increases from 1.39 to 1.40. When the analyte RI changes from 1.32 to 1.41 in steps of 0.01, the corresponding amplitude sensitivity is 136.67, 152.05, 140.25, 197.70, 208.32, 263.07, 257.38, 354.39, and 339.80 RIU^−1^, respectively.

Another important index FOM is calculated in our work. FOM indicates the sharpness of the CL peak and evaluates the detecting accuracy. FOM is defined as [28]:

(11) $$\mathrm{FOM}\left({\mathrm{RIU}}^{-1}\right)=\frac{S_{\lambda }\left(n_{a},\lambda \right)}{\mathrm{FWHM}\left(\mathrm{nm}\right)}$$

where $S_{\lambda }\left(n_{a},\lambda \right)$ is the wavelength sensitivity. $\mathrm{FWHM}\left(\mathrm{nm}\right)$ denotes the full width at half maxima. FOM combines FWHM and signal-to-noise ratio (SNR). For higher sensitivity, the FOM is required to be as large as possible. In Figure 5b, an ideal FOM of 216.74 RIU^−1^ is obtained at analyte RI = 1.40.

### 3.2. Temperature Sensing

**Figure 6:**
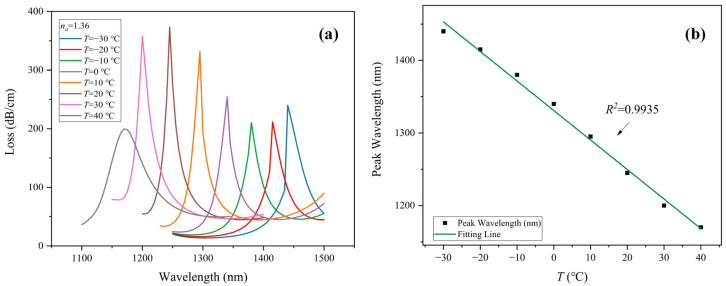
(**a**) CL spectrum of *x*-polarization core mode under various *T* from −30–40 °C when *n_a_* = 1.36. (**b**) Resonance wavelength and its linear fitting line under various *T* from −30–40 °C when *n_a_* = 1.36.

Figure 6a shows the loss spectrum of the PCF-SPR sensor at −30–40 °C. Analyte RI is fixed at 1.36. Corresponding to a temperature of −30, −20, −10, 0, 10, 20, 30, and 40 °C, SPR appears at a wavelength of 1440, 1415, 1380, 1340, 1295, 1245, 1200, and 1170 nm, respectively. As the temperature increases, the resonance wavelength shows a blue-shift towards a smaller wavelength, for the RI of liquid toluene becomes smaller as the temperature increases. The SPR occurring at the surface of the gold layer correspondingly shifts to a shorter wavelength with increasing temperature. The wavelength sensitivity of temperature detection is calculated as follows [29]:

(12) $$S_{\lambda }\left(T,\lambda \right)=\frac{\Delta \lambda_{\mathrm{peak}}}{\Delta T}\left(\mathrm{nm}/{}^{\circ}C\right)$$

where $\Delta \lambda_{\mathrm{peak}}$ refers to the shift of peak wavelength under adjacent temperature *T*. The variant of temperature is denoted by ΔT. The proposed sensor achieves wavelength sensitivity of −2.5, −3.5, −4.0, −4.5, −5.0, −4.5, and −3.0 nm/°C for the environment temperature varying from −30 °C to 40 °C in steps of 10 °C, respectively. The best sensitivity of −5.0 nm/°C is obtained as temperature diverse from 10 °C to 20 °C. As shown in Figure 6b, the fitting result of the resonance wavelength shows good linearity with an excellent *R*^2^ of 0.9935.

The resolution of temperature is defined as [30]:

(13) $$R_{T}\left(T,\lambda \right)=\Delta T\times \Delta \lambda_{\min}/\Delta \lambda_{\mathrm{peak}}\left({}^{\circ}C\right)$$

where ΔT is the change of the ambient temperature, $\Delta \lambda_{\min}$ denotes the smallest spectrum resolution, which is also set as 0.1 nm. $\Delta \lambda_{\mathrm{peak}}$ refers to the shift wavelength of the resonance peak when temperature varies. According to Equation (13), the resolution is 0.02 °C when the temperature changes from 10 °C to 20 °C. It is worth mentioning that the reason we choose −30–40 °C as the temperature detecting range is for the consideration of both toluene characteristics and sensing performance. The melting and boiling point of liquid toluene is −94.9 °C and 110.6 °C, respectively [31]. Toluene remains chemically stable in this temperature range when sealed. Additionally, the reported sensor shows good sensitivity and peak wavelength linearity within the ambient temperature range from −30 °C to 40 °C.

### 3.3. Independence Analysis of Dual Parameter Sensing

**Figure 7:**
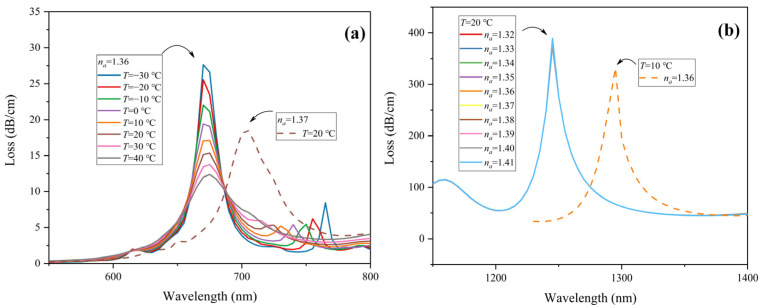
(**a**) CL spectrum of *y*-polarization core mode in RI sensing channel under different ambient temperatures *T* from −30 to 40 °C when *n_a_* = 1.36 (solid line) and under *T* = 20 °C when *n_a_* = 1.37 (dotted line). (**b**) CL spectrum of *x*-polarization core mode in temperature sensing channel for various *n_a_* from 1.32–1.41 at *T* = 20 °C (solid line) and under *T* = 10 °C when *n_a_* = 1.36 (dotted line).

The crosstalk between the two sensing channels should be considered in dual-parameter measurement sensors. In this part, we analyze the performance in the two sensing channels under different temperatures and analyte RI. Firstly, the analyte RI is fixed at 1.36 with the ambient temperature varying from −30 °C to 40 °C. The CL spectrum of the RI sensing channel is drawn in Figure 7a. As the temperature alters, core mode loss in *y*-polarization only has a slight change in amplitude, while the resonance wavelength barely changes and is fixed at 675 nm. For comparison, the CL spectrum of the *y*-polarization core mode when the analyte RI is 1.37 at 20 °C is also plotted in Figure 7a. The core mode loss in *y*-polarization has a greater change in amplitude; the peak wavelength also red-shifts from 675 nm to 705 nm. Therefore, the RI sensing region is insensitive to temperature. Next, the ambient temperature remains constant at 20 °C; the analyte RI is modified from 1.32 to 1.41. As can be seen in Figure 7b, the core mode loss in *x*-polarization stays the same. Similarly, when the analyte RI is 1.36 and the ambient temperature is 10 °C, the loss spectrum of the *x*-polarization core mode is depicted in Figure 7b. It can be found that the peak wavelength red-shifts from 1245 nm to 1295 nm. Therefore, the temperature sensing channel is insensitive to the analyte RI. Given the above, the RI sensing channel and temperature sensing channel are independent during measurement, which helps avoid sensitivity crossover and simplifies the detecting process.

## 4. Structure Parameter Optimization

The structure parameters of the sensor will affect the detection performance of temperature and RI in various degrees. In order to optimize the sensing characteristics of the proposed sensor, the influence of the thickness of the gold film *t_g_*, deposition depth of the gold film *h*, the diameter of the small air holes in the inner layer *d_s_*, and the diameter of the large air holes in the outer layer *d_l_* on the sensing performance are analyzed and discussed in this section.

**Figure 8:**
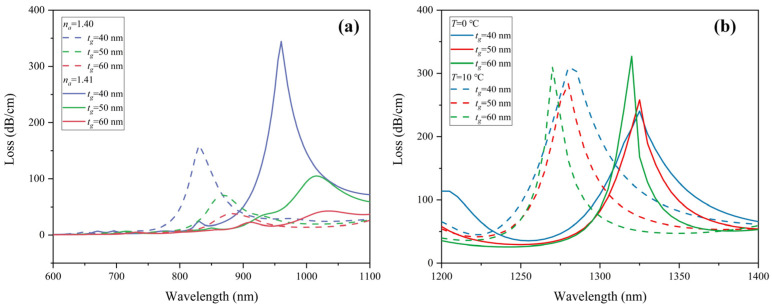
Loss spectra of (**a**) *y*-polarization core mode in RI sensing channel when analyte RI varies from 1.40 to 1.41, and (**b**) *x*-polarization core mode in temperature sensing channel under ambient temperature from 0 to 10 °C with different thicknesses of the Au film *t_g_*.

SPR is excited at the interface between the gold layer and the medium. The thickness of the gold layer *t_g_* has a great influence on the resonance wavelength and the peak value of the loss spectrum. Therefore, this parameter is one of the key indicators that affect the sensing performance. It can be seen from Figure 8a that the increase in *t_g_* promotes the red-shift of the resonance wavelength. When *t_g_* = 40, 50, and 60 nm, the wavelength sensitivity of RI is 13,000, 14,500, and 15,500 nm/RIU, respectively. As *t_g_* increases, the height of the loss peak decreases. This is because the gold film is too thick, preventing the electric field from penetrating the dielectric layer [32]. The mode coupling efficiency is correspondingly depressed and weakened, resulting in great suppression of the CL peak. Additionally, the CL spectrum of 1.41 RIU shows a 2nd order peak near the resonance peak of 1.40 RIU when *t_g_* = 60 nm, which might interfere with the sensing. For temperature detection, as shown in Figure 8b, when *t_g_* = 40 and 50 nm, the wavelength sensitivity is −4.5 nm/°C, and when *t_g_* thickens to 60 nm, the temperature sensitivity is increased to −5 nm/°C. Considering the sensing characteristics of both RI and temperature, 50 nm is determined to be the optimum thickness of the gold film.

**Figure 9:**
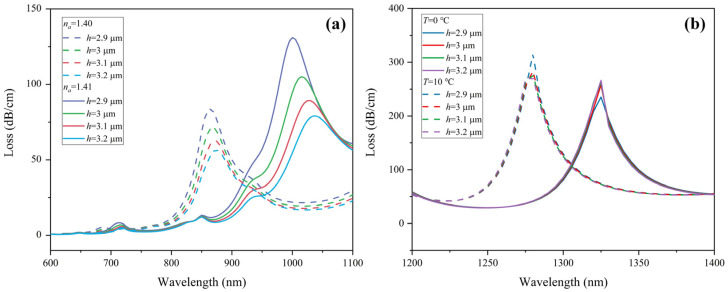
Loss spectra of (**a**) *y*-polarization core mode in RI sensing channel when analyte RI varies from 1.40 to 1.41, and (**b**) *x*-polarization core mode in temperature sensing channel under ambient temperatures from 0 to 10 °C with different polishing depths of Au film *h*.

Since the detection of RI occurs in *y*-polarization, the polishing depth *h* of the Au film has a great influence on the sensing performance of RI. Figure 9a exhibits the CL spectra of 1.40 RIU and 1.41 RIU as a function of *h*. When the analyte RI varies from 1.40 to 1.41, the corresponding wavelength sensitivities are 13,500, 14,500, 16,000, and 16,000 nm/RIU for *h* = 2.9, 3, 3.1, and 3.2 μm, respectively. When *h* is larger than 3.1 μm, the sensitivity is no longer improved. It can be seen from Figure 9a that the CL peak becomes lower as *h* increases. As the polishing surface gets away from the core area, resonance intensity between the core mode and SPP mode is weakened accordingly. Figure 9b shows the resonance wavelength as a function of different *h* at 0–10 °C. It is obvious that the change in *h* has little effect on temperature sensitivity, which remains at −4.5 nm/°C. Therefore, we choose *h* = 3.1 μm for higher RI sensitivity.

**Figure 10:**
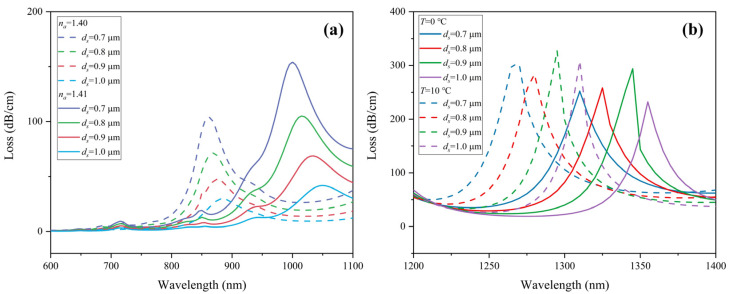
Loss spectra of (**a**) *y*-polarization core mode in RI sensing channel when analyte RI varies from 1.40–1.41, and (**b**) *x*-polarization core mode in temperature sensing channel under ambient temperature from 0–10 °C with different diameter of air holes *d_s_*.

The structure of PCF can be further optimized. The diameter of the air holes has a great influence on the leakage of the mode and the position where the resonance occurs. Firstly, the influence of the small air hole diameter *d_s_* in the inner layer on sensing performance is analyzed. Figure 10a shows the effect of *d_s_* on the CL spectrum when the analyte RI changes from 1.40 to 1.41. When *d_s_* = 0.7, 0.8, 0.9, and 1 μm, the RI sensitivity is 14,000, 14,500, 16,000, and 18,500 nm/RIU, respectively. It can be known that smaller *d_s_* allows the guiding light to leak more from the core area, resulting in a stronger resonance at the interface between the metal film and the medium. A larger peak in the CL spectrum appears. Red-shift of the resonance wavelength is suppressed, and the RI sensitivity is reduced. From Figure 10b, when *d_s_* = 0.7, 0.8, 0.9, and 1 μm, the temperature sensitivity is −3.5, −4.5, −5, and −4.5 nm/°C, respectively. As *d_s_* increases from 0.7 μm to 0.9 μm, the temperature sensitivity ascends. However, when *d_s_* continues to increase, the temperature sensitivity begins to descend afterward. Therefore, the value of *d_s_* is set as 0.9 μm.

**Figure 11:**
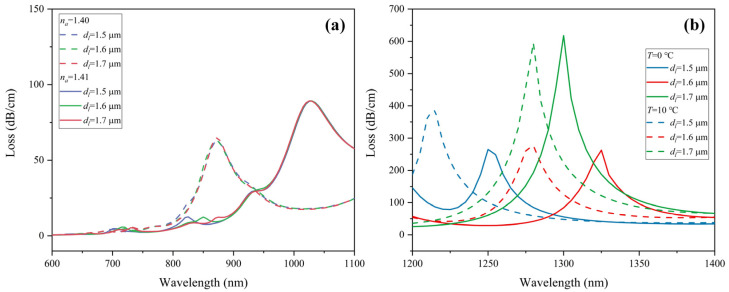
Loss spectra of (**a**) *y*-polarization core mode in RI sensing channel when analyte RI varies from 1.40 to 1.41, and (**b**) *x*-polarization core mode in temperature sensing channel under ambient temperature from 0 to 10 °C with different diameter of air holes *d_l_*.

Figure 11 displays the variations of the loss spectrum with a different air hole diameter *d_l_* for the RI range 1.40–1.41 and the temperature range 0–10 °C. Because there is no large air hole between the RI detection channel and fiber core area, the variation of *d_l_* does not have much influence on the RI detection, yet it has a certain influence on the temperature detection. Figure 11a shows that when *d_l_* = 1.5, 1.6, and 1.7 μm, the RI sensitivity is 15,500, 16,000, and 15,000 nm/RIU, respectively. Figure 11b shows that the corresponding temperature sensitivity is −3.5, −4.5, and −2 nm/°C, respectively. When *d_l_* = 1.6 μm, both RI and temperature detection have better performance. Thus, *d_l_* = 1.6 μm is considered the best design. In conclusion, the optimized structural parameters are *d_s_* = 0.9 μm, *d_l_* = 1.6 μm, *h* = 3.1 μm, and *t_g_* = 50 nm.

Table 1 illustrates the comparison of the sensing characteristics between the proposed flat PCF-SPR sensor and previously reported structures for RI and temperature detection. Compared with other structures, it is evident that the detecting performance of temperature and RI is greatly improved in the proposed PCF. The sensor shows more superb wavelength sensitivity of 17,000 nm/RIU and amplitude sensitivity of 354.39 RIU^−1^ for RI sensing than the previously reported sensors. The optimal temperature sensitivity of −5 nm/°C is comparable. In addition, the proposed structure is simpler than some of the structures that have been introduced.

## 5. Fabrication Tolerance and Manufacturing Process Analysis

In the practical manufacturing process, the difference in structure parameters is inevitable. According to Reeves et al. [36], the order of 1% deviation might take place. In this section, the repeatability and stability of the proposed sensor are investigated by performing fabrication tolerance on the key parameters *t_g_*, *h*, *d_s_*, and *d_l_*.

**Figure 12:**
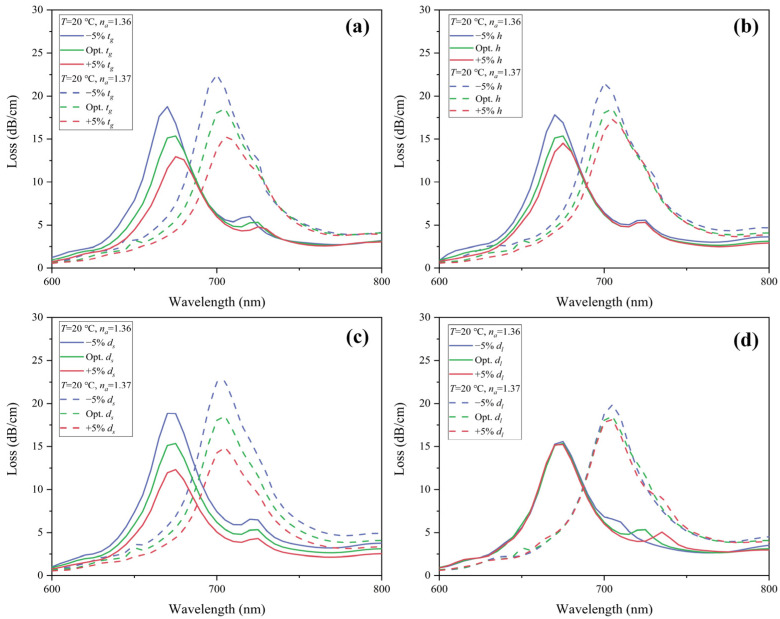
Tolerance test on RI sensing channel of the structure parameter (**a**) *t_g_*, (**b**) *h*, (**c**) *d_s_*, and (**d**) *d_l_* for ±5% deviation.

**Figure 13:**
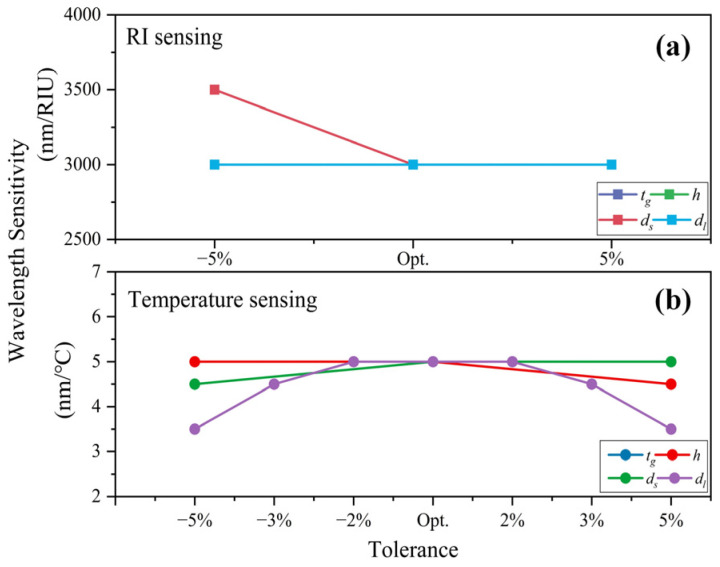
Wavelength sensitivity of (**a**) RI sensing for structure parameter *t_g_*, *h*, *d_s_*, and *d_l_* with ±5% tolerance, and (**b**) temperature sensing for structure parameter *t_g_*, *h*, *d_s_* with ±5% tolerance, and *d_l_* with ±2%, ±3%, ±5% tolerance.

We consider ±5% variation from the optimal parameters of *t_g_*, *h*, *d_s_*, and *d_l_*. Under ambient temperature *T* = 20 °C, the CL spectra of *y*-polarization core mode in the RI sensing channel for analyte RI of 1.36 and 1.37 is depicted in Figure 12. Figure 12a–d shows the result of the tolerance test for *t_g_*, *h*, *d_s_*, and *d_l_*, respectively. The resonance peak shift is less than 5 nm, which is very minor. The change in peak value is very slight and is under 4.12 dB/cm. Figure 13a shows the wavelength sensitivity of RI detection with ±5% structure tolerance. It is obvious that except for an improvement of 500 nm/RIU with −5% *d_s_*, wavelength sensitivity remains unchanged. Therefore, ±5% tolerance of the structure parameters has little effect on RI sensing.

**Figure 14:**
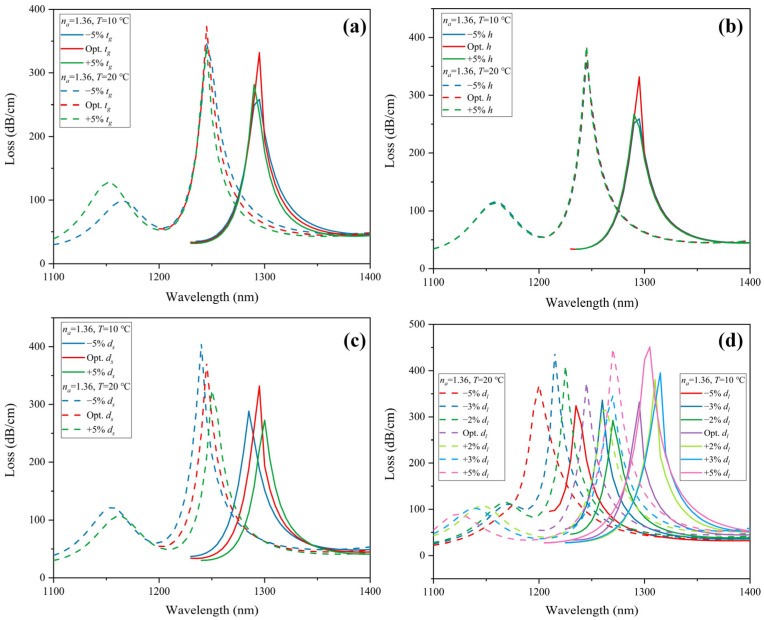
Tolerance test on temperature sensing channel of the structure parameter (**a**) *t_g_*, (**b**) *h*, (**c**) *d_s_* for ±5% deviation, and (**d**) *d_l_* for ±2%, ±3%, ±5% deviation.

A tolerance test on structure parameters is also performed in the temperature-sensing channel. A ±5% deviation of *t_g_*, *h*, *d_s_*, *d_l_*, and ±2%, ±3%, and ±5% deviation of *d_l_* are considered. With analyte RI of 1.36, the CL spectrum of the *x*-polarization core mode under ambient temperature *T* = 10 and 20 °C is drawn in Figure 14. Figure 14a–d shows the result of the tolerance test for *t_g_*, *h*, *d_s_*, and *d_l_*, respectively. *t_g_*, *h,* and *d_s_* with ±5% deviation did not show a significant effect on wavelength shift, which is less than 5 nm. The change in peak value of CL spectra is under 73.46 dB/cm. In Figure 13b, the decrease in wavelength sensitivity for temperature sensing caused by *t_g_*, *h,* and *d_s_* with ±5% deviation is less than 0.5 nm/°C. However, ±5% deviation of *d_l_* will have a greater effect on both wavelength shift and peak value. Thus, we further examine the temperature sensing characteristics with ±2% and ±3% tolerance of *d_l_*. As shown in Figure 14d, with ±3% deviation of *d_l_*, the corresponding maximum wavelength shift is 35 nm, and the maximum peak loss change is 65.96 dB/cm. With ±2% deviation of *d_l_*, the wavelength shift is 25 nm and 20 nm for −2% under *T* = 10 and 20 °C, respectively, and 15 nm for +2% under *T* = 10 and 20 °C. The maximum peak loss change is 58.17 dB/cm. From Figure 13b, with ±3% tolerance of *d_l_*, the variation of wavelength sensitivity for temperature sensing can be limited within 0.5 nm/°C. With ±2% tolerance of *d_l_*, the wavelength sensitivity is unaffected. In order to ensure the loss peak shift is as minor as possible, we suggest the fabrication tolerance of *d_l_* be controlled under ±2%, which is technically feasible. Overall, the reported sensor exhibits considerable repeatability and stability when the structure parameters *t_g_*, *h*, and *d_s_* are fabricated within ±5% tolerance and *d_l_* within ±2% tolerance. Moreover, gold is very stable. Liquid toluene and gold are chemically nonreactive. As an organic compound, toluene does not corrode silica. Since toluene is packaged in the PCF, volatility can be avoided. The material and structure design helps the sensor stay stable during the detecting process.

**Figure 15:**
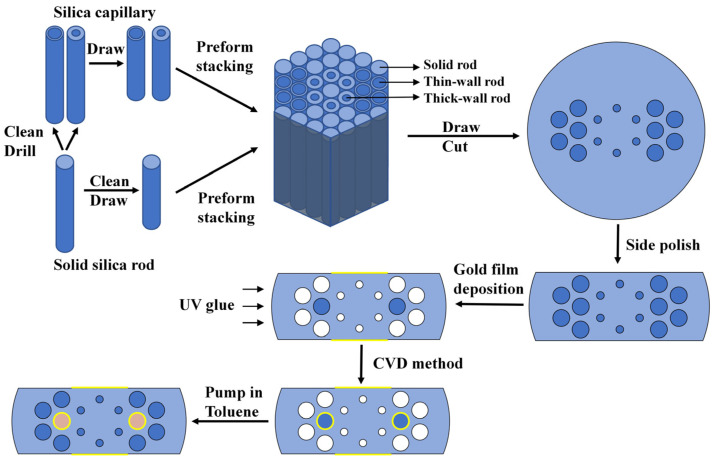
Manufacturing process of the proposed flat PCF plasmonic sensor.

Manufacturing is an essential part of the proposed sensor to be practically applied. In Figure 15, the manufacturing process of the reported flat PCF plasmonic sensor is illustrated. We suggest the following ways by which our sensor can be implemented. The stack-and-draw method [37] can be adopted to fabricate the PCF structure. Silica tubes with good geometric dimensions and optical surfaces are selected. Capillaries of different diameters are drawn through a drawing tower after rigorous cleaning. The drawn capillaries are tightly packed according to the designed structure. The preform stacking configuration of the proposed sensor is shown in Figure 15. Solid rods, thick-wall, and thin-wall rods are used to form cavities of different sizes. The stacked rods are drawn in a jacket through a high-temperature furnace that is heated to 1850–1900 °C [38] to soften. The cane is cut into the desired length. Using the side polishing method [39], the gold coating surface is produced. The gold film is then coated on the PCF. The deposition process can be realized by chemical vapor deposition (CVD) [40], atomic layer deposition (ALD) [41], and other methods [42]. Next, air holes, except for the selected liquid-filled ones, are sealed with UV glue. UV glue is hardened under a UV radiator. The gold film on the inner air holes can also be deposited by the aforementioned methods. Liquid toluene is then pumped into the selected air holes in a vacuum [43]. In the next step, the experimental realization of the proposed work is expected to examine and improve our structure.

## 6. Conclusions

In this paper, a sensitive and compact RI-temperature PCF sensor based on SPR is presented. The flat RI detection channel is coated with gold film. The temperature detection channel is composed of two liquid toluene-filled air holes and incorporated with a gold film. The large thermos-optical coefficient of toluene improves the temperature sensitivity. The detection of temperature and RI takes place in the *x*- and *y*- polarization, respectively, to avoid the detection crossover. By optimizing the structural parameters of the sensor, the detection sensitivity is improved. A maximum wavelength sensitivity of −5 nm/°C is achieved in the temperature range of −30–40 °C. An excellent optimal wavelength sensitivity of 17,000 nm/RIU is accomplished in the RI range from 1.32 to 1.41. The simulation results show that the proposed sensor possesses higher sensitivity than the previously reported structures. For temperature sensing, the fitting result of the resonance wavelength shows good linearity with an excellent *R*^2^ of 0.9935. For RI sensing, polynomial fitting of the resonance wavelength also gives the extremely high fitting coefficient *R*^2^ of 0.99896. The proposed sensor shows an ideal detecting resolution of 5.88 × 10^−6^ RIU and 0.02 °C for RI and temperature, respectively. The best amplitude sensitivity can be up to 354.39 RIU^−1^. The best value of FOM is 216.74 RIU^−1^. The sensor shows good repeatability when *t_g_*, *h*, and *d_s_* are fabricated within ±5% tolerance and *d_l_* within ±2% tolerance. In addition, the proposed sensor structure can be easily manufactured. The flat polishing area of the gold layer reduces the manufacturing difficulty. Due to the characteristics of high sensitivity, simple structure, and flexible operation, the novel flat PCF sensor has a promising future in environmental monitoring, chemical inspection, and many other fields.

## Figures and Tables

**Table 1:** Performance comparison between the proposed sensor and previously reported sensors.

Structure Configuration	RI Range	Temp. Range	Operating Wavelength [nm]	Wavelength Sensitivity (RI/Temp.)	Amplitude Sensitivity (RI/Temp.) [RIU^−1^/°C^−1^]	Wavelength Resolution (RI/Temp.) [RIU/°C]	FOM (RI) [RIU^−1^]
Liquid-filled D-shape PCF [12]	1.35–1.40	20–60 °C	550–850	3940 nm/RIU/ 1.075 nm/°C	152.23/ 539.42	N/A	N/A
No-core fiber [13]	1.33–1.44	0–180 °C	400–1200	12,530 nm/RIU/ −3.465 nm/°C	N/A	N/A	N/A
Double U-groove PCF [33]	1.32–1.4	−30–50 °C	1300–2500	4715 nm/RIU/ 18 nm/°C	48.44/ 1.01 × 10^−1^	2.12 × 10^−5^/ 5.55 × 10^−3^	N/A
Dual-core D-shaped PCF [34]	1.33–1.39	−50–40 °C	500–900	8100 nm/RIU/ 1.3 nm/°C	N/A	N/A	N/A
Grating-assisted SPR silicon core sensor [35]	1.28–1.38	15–40 °C	1800–2800	1949.8 nm/RIU/ 1.6 nm/°C	N/A	N/A	N/A
Proposed Flat PCF Plasmonic Sensor	1.32–1.41	−30–40 °C	500–1500	17,000 nm/RIU/ −5 nm/°C	354.39/ N/A	5.88 × 10^−6^/ 0.02	216.74

## Data Availability

Not applicable.
